# Comparative Efficacy of Tulathromycin and Ceftiofur for Treating Undifferentiated BRDC and Tulathromycin Metaphylaxis in Dairy Cattle

**DOI:** 10.3390/antibiotics15020154

**Published:** 2026-02-02

**Authors:** Sahaphop Pengpanun, Surachet Panyapan, Tawatchai Singhla

**Affiliations:** 1 Faculty of Veterinary Medicine, Chiang Mai University, Chiang Mai 50100, Thailand; sahaphop_p@cmu.ac.th (S.P.); surachet.p@cmu.ac.th (S.P.); 2 Research Center for Veterinary Biosciences and Veterinary Public Health, Faculty of Veterinary Medicine, Chiang Mai University, Chiang Mai 50100, Thailand

**Keywords:** bovine respiratory disease complex, tulathromycin, ceftiofur, dairy cattle

## Abstract

Background/Objectives: Bovine respiratory disease complex (BRDC) is commonly treated empirically, as etiological diagnosis is often impractical under field conditions. Comparative evidence on antimicrobial efficacy in undifferentiated BRDC remains limited. This study aimed to compare the therapeutic efficacy of tulathromycin and ceftiofur for treating undifferentiated BRDC in dairy cattle and to describe hematological and biochemical responses following a tulathromycin metaphylaxis program implemented during the seasonal high-PM2.5 period in northern Thailand. Methods: Thirty-eight Holstein–Friesian cattle with clinical BRDC were randomly assigned to receive tulathromycin (2.5 mg/kg, single subcutaneous dose; *n* = 20) or ceftiofur (2.2 mg/kg, intramuscularly for three consecutive days; *n* = 18). The clinical parameters of the surviving cattle were monitored for 5 days, and hematological and biochemical profiles were assessed on Days 1 and 5 in the surviving cattle. In addition, 87 pregnant dairy heifers were enrolled in a metaphylaxis trial and allocated to no injection, one tulathromycin injection, or two injections administered one month apart. Results: Cure rates were comparable between the tulathromycin and ceftiofur groups (90.0% vs. 88.9%), with similar case fatality rates (10.0% vs. 11.1%). No significant between-group differences were observed for clinical, hematological, or biochemical parameters. Both treatments resulted in significant within-group clinical and hematological improvement. During the metaphylaxis trial, no animals developed clinical BRDC; however, significant differences were observed in selected hematological parameters among injection groups. Conclusions: Tulathromycin and ceftiofur demonstrated comparable efficacy for treating undifferentiated BRDC in dairy cattle under field conditions. In the metaphylaxis component, conducted during the seasonal high-PM2.5 period, the absence of clinical BRDC cases did not allow for evaluation of preventive efficacy. Nevertheless, differences in selected blood parameters were observed among injection groups and should be interpreted cautiously, warranting further investigation in studies incorporating low- and high-PM2.5 comparisons.

## 1. Introduction

Bovine respiratory disease complex (BRDC) is one of the most economically important diseases affecting the global cattle industry, leading to substantial losses from decreased growth performance, milk yield reduction, treatment expenses, and mortality, with annual economic losses estimated at approximately $800 million [1,2,3]. In dairy herds, respiratory disease is a major cause of morbidity and treatment, particularly in calves and heifers, and is increasingly recognized in adult cattle [1,2]. In pre-weaned calves, it accounts for 14.1% of deaths, with the reported BRDC prevalence ranging from 4% to 80% [4]. The pathogenesis of BRDC is multifactorial, involving interactions among viral pathogens such as bovine herpesvirus-1, bovine respiratory syncytial virus, parainfluenza virus type 3, bovine viral diarrhea virus, and bovine coronavirus; bacterial agents such as *Mannheimia haemolytica* (*M. haemolytica*), *Pasteurella multocida* (*P. multocida*), *Histophilus somni* (*H. somni*), and *Mycoplasma bovis* (*M. bovis*); host immunity; environmental stressors; and management practices [5,6,7,8,9,10].

The success of BRDC treatment depends on its early detection and the timely use of appropriate antibiotics and anti-inflammatory drugs [11,12]. In field conditions, however, the precise etiological diagnosis of BRDC prior to treatment is often impractical. The acute onset of clinical signs such as fever, nasal discharge, coughing, dyspnea, and reduced appetite frequently prompts immediate therapy to prevent irreversible lung damage and further transmission within the herd [11,12]. Laboratory confirmation of pathogens requires time, specialized sampling, and diagnostic facilities, which are rarely available at the onset of clinical disease [13]. Consequently, BRDC treatments often rely on empirical antimicrobial therapy based on clinical presentation and herd history rather than pathogen identification, especially in resource-poor settings [14].

This empirical approach is justified by the urgency of alleviating animal suffering and reducing disease progression, as delays in treatment have been associated with poorer outcomes and higher mortality [15]. However, it also raises concerns about appropriate antimicrobial selection, potential overuse or misuse, and the emergence of antimicrobial resistance [16,17]. Therefore, evidence-based comparisons of commonly used antibiotics in cases of undifferentiated pneumonia, where the causative agent remains unknown, are critical for guiding rational treatment strategies.

Among the antimicrobial agents approved for BRDC, tulathromycin and ceftiofur are two widely used options with different pharmacological properties. Tulathromycin, a long-acting macrolide, demonstrates high tissue penetration and prolonged activity against key BRDC pathogens, including *M. haemolytica*, *P. multocida*, *H. somni*, and *M. bovis* [18,19]. Ceftiofur, a third-generation cephalosporin, is valued for its broad-spectrum activity, safety profile, and short withdrawal period, making it suitable for lactating dairy cattle [20,21]. Although both drugs are registered for BRDC treatment, comparative data under field conditions, particularly when treatment is initiated without prior etiologic confirmation, are still scarce. Therefore, the present study aimed to compare the therapeutic efficacy of tulathromycin and ceftiofur in the treatment of undifferentiated pneumonia (BRDC) in dairy cattle in Thai dairy herds.

Air pollution remains a major global environmental challenge, and fine particulate matter (PM2.5; aerodynamic diameter ≤ 2.5 µm) is recognized as one of its most harmful components for both humans and animals [22,23,24]. Experimental evidence in animal models indicates that PM2.5 induces respiratory inflammation, disrupts the respiratory microbiota, and contributes to the development of respiratory disease [25]. PM2.5 exposure has also been associated with alterations in cytokine responses, impaired pulmonary function, coagulation imbalance, and cardiovascular dysfunction [26].

Thailand experiences seasonal PM2.5 episodes, with northern provinces such as Chiang Mai frequently recording elevated concentrations during the annual haze period [27]. Respiratory disease burden in cattle, including BRDC, has been reported to increase during periods of heightened environmental stress in the region [28]. However, the extent to which PM2.5 specifically influences BRDC occurrence or clinical outcomes under field conditions remains uncertain.

Considering these challenges, prevention and timely treatment of BRDC are critical. Several studies have demonstrated the benefits of metaphylactic antibiotic administration during periods of stress or immunosuppression. In feedlot and stocker operations, tulathromycin administered at arrival or before transportation significantly reduced BRDC morbidity and mortality in high-risk calves exposed to stressors such as long-distance transport, commingling, and crowding [29,30,31,32]. Nevertheless, evidence supporting metaphylaxis in dairy cattle under naturally occurring seasonal conditions is limited, and data in dairy heifers remain scarce.

Therefore, the present study aimed (i) to compare the therapeutic efficacy of tulathromycin and ceftiofur for the treatment of undifferentiated bovine respiratory diseases in dairy heifers, and (ii) to evaluate the efficacy of tulathromycin metaphylaxis for preventing bovine respiratory diseases in pregnant dairy heifers during the high-PM2.5 season in Chiang Mai, Thailand.

## 2. Results

### 2.1. Comparison of Therapeutic Efficacy Between Tulathromycin and Ceftiofur

#### 2.1.1. Baseline Characteristics

A total of 38 Holstein–Friesian dairy cattle diagnosed with undifferentiated BRDC were enrolled in the treatment comparison, including 20 animals treated with tulathromycin and 18 treated with ceftiofur. Baseline demographic characteristics, clinical variables, and physiological parameters are summarized in Table 1. No significant differences were observed between treatment groups with respect to age, body weight, rectal temperature (RT), respiratory rate (RR), ruminal contraction rate (RCR), or clinical attitude scores (CAS) at enrollment (*p* > 0.05). The distribution of major respiratory clinical signs, including fever, nasal discharge, coughing, anorexia, and abnormal locomotion, was also comparable between groups, indicating similar disease severity at baseline.

#### 2.1.2. Clinical Outcomes and Treatment Efficacy

**Figure 1:**
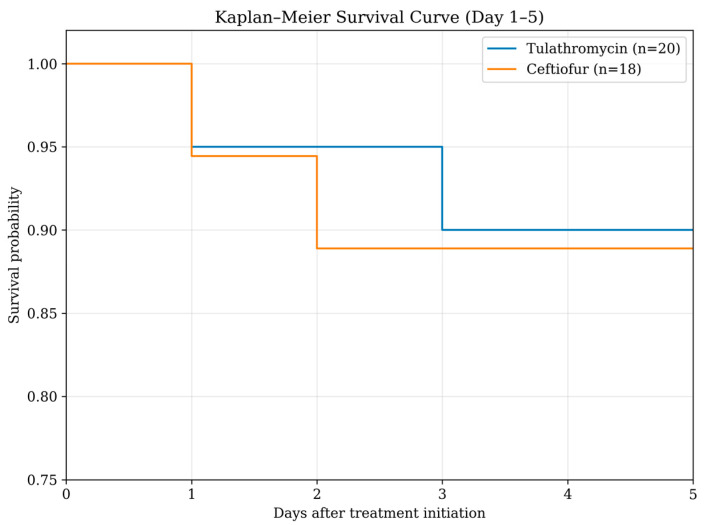
Kaplan–Meier survival curves of dairy cattle treated with tulathromycin (*n* = 20) or ceftiofur (*n* = 18) during the 5-day follow-up period.

The case fatality rate was 10.0% (2/20) in the tulathromycin group and 11.1% (2/18) in the ceftiofur group. Correspondingly, cure rates were 90.0% (18/20) and 88.9% (16/18), respectively. In both treatment groups, one animal died on Day 2 and another on Day 3 following initiation of therapy. Therefore, clinical outcome analyses were conducted in 18 and 16 cattle in the tulathromycin and ceftiofur groups, respectively. Kaplan–Meier survival analysis demonstrated comparable survival patterns between the tulathromycin and ceftiofur groups during the 5-day follow-up (Figure 1). In the tulathromycin treatment group, the fatal cattle were 3 months and 2 years of age. The 3-month-old calf showed clinical signs of dyspnea and sternal recumbency, while the other animal showed dyspnea only. In the ceftiofur treatment group, one fatal calf was 5 months of age and showed dyspnea and increased respiratory effort. Another fatal animal was 3 years of age and presented with subcutaneous emphysema and dyspnea. As a result, the number of animals included in the comparisons on days 1 and 5 decreased following fatalities in each treatment group (tulathromycin, *n* = 18; ceftiofur, *n* = 16).

No significant differences were observed between treatment groups including RCR, RR, RT, coughing, nasal discharge, appetite, locomotion, and CAS at any time point from Day 2 to Day 5 as shown in Supplementary Table S1. However, within-group analyses demonstrated significant clinical improvement in both treatment groups. From Day 1 to Day 5, cattle treated with either tulathromycin or ceftiofur showed significant reductions in RT and RR, along with marked improvements in RCR, nasal discharge, appetite, locomotion, and CAS (*p* < 0.05) as shown in Table 2.

#### 2.1.3. Hematological and Biochemical Responses to Treatment

Hematological and biochemical parameters measured on Days 1 and 5 are presented in Table 3. In the tulathromycin-treated group, significant decreases were observed between Day 1 and Day 5 in total white blood cell (WBC), neutrophil (Neu), red blood cell (RBC), hemoglobin (Hb), hematocrit (HCT), blood urea nitrogen (BUN), creatinine, and Alkaline Phosphatase (ALP) (*p* < 0.05). Lymphocyte count (Lymp) and liver enzyme activities (aspartate aminotransferase [AST] and Alanine Aminotransferase [ALT]) did not change significantly over time (*p* > 0.05).

Similarly, in the ceftiofur-treated group, significant reductions were detected in total WBC, RBC, Hb, HCT, BUN, creatinine, AST, and ALT between Day 1 and Day 5 (*p* < 0.05). Changes in Neu, Lymp, and ALP activity were not statistically significant (*p* > 0.05). However, no significant between-group differences were identified for any hematological or biochemical parameter at either sampling time point (*p* > 0.05) as shown in Supplementary Table S2.

### 2.2. Tulathromycin Metaphylaxis During High-PM2.5 Period

#### 2.2.1. Clinical Outcomes

Throughout the study period, no cattle in any of the three groups (no injection, one-time injection, or two-times injection) developed clinical signs of bovine respiratory disease complex during the experimental period, and no morbidity was observed.

#### 2.2.2. Hematological and Biochemical Responses to Treatment

Results of generalized estimating equation analysis are summarized in Table 4, with the complete dataset provided in Supplementary Table S3. No significant effects of injection regimen, blood collection time (before vs. after injections), or their interaction were observed for WBC, Lymp, monocytes, eosinophils or RBC (all *p* > 0.05). In contrast, Neu differed significantly among injection groups (group effect *p* = 0.01), with numerically higher values in the no-injection group at both time points compared with the one-injection and two-injection groups. For erythrocyte indices, Hb and HCT were significantly influenced by injection group and the time × group interaction (both group *p* < 0.001; interaction *p* ≤ 0.001). Notably, HB and HCT decreased from before to after injections in the no-injection group, while they were maintained or increased in the injection groups, particularly in the two-injection group. Platelet count increased from before to after injections across groups (time effect *p* = 0.002).

Serum biochemistry profiles showed no significant group, time, or interaction effects for BUN (*p* > 0.05) with mean values remaining low across all groups. Creatinine also showed no significant model effects (*p* > 0.05), with stable mean values at both time points across regimens. Total protein demonstrated a significant time × group interaction (*p* = 0.006), reflecting different temporal patterns among groups. Albumin showed a modest time effect (*p* = 0.04) with a small decrease in the no-injection group, while remaining stable in the injection groups. ALT did not differ significantly by group, time, or their interaction (*p* > 0.05), with values remaining within a comparable range across regimens.

## 3. Discussion

This study demonstrated that tulathromycin and ceftiofur provided comparable therapeutic efficacy for the treatment of undifferentiated BRDC in dairy cattle under field conditions in northern Thailand.

Cure rates were similarly high in both groups (tulathromycin = 90.0% and ceftiofur = 88.9%), and case fatality rates were low and comparable (10.0% vs. 11.1%). Mortality occurred early during treatment (Days 2–3) and was associated with severe clinical presentations at enrollment, consistent with previous reports indicating that antimicrobial therapy alone may be insufficient in advanced or multifactorial BRDC cases [33,34,35,36].

The observed mortality rates fall within the range reported in earlier studies of BRDC treatment using macrolides and cephalosporins, although wide variation has been documented depending on disease severity, management conditions, and population characteristics [34,35,36]. However, necropsy examinations were not performed for the animals that died during the study, as the trial was conducted under field conditions in commercial dairy farms where post-mortem investigation was not feasible. Although these animals were clinically diagnosed with BRDC prior to death, the cause of death could not be definitively confirmed. Therefore, the reported case fatality rate should be interpreted with caution. Collectively, these findings are consistent with previous reports indicating that both tulathromycin and ceftiofur are effective therapeutic options for BRDC under field conditions.

Between-group comparisons revealed no significant differences between tulathromycin-and ceftiofur-treated cattle for any evaluated clinical parameter from Day 2 to Day 5, including RT, RR, RCR, coughing, nasal discharge, appetite, locomotion, and CAS. These results indicate equivalent clinical responses between treatment groups, consistent with previous comparative studies evaluating antimicrobial efficacy in BRDC under field conditions [37,38,39].

Despite the absence of between-group differences, both antimicrobials produced significant within-group improvements. Reductions in RT and RR, alongside marked improvements in RCR and CAS, reflect effective resolution of systemic inflammation and respiratory compromise. The decline in RT observed in both groups is biologically plausible given the broad antimicrobial activity of tulathromycin against *M. haemolytica*, *P. multocida*, *H. somni*, and *M. bovis*, and of ceftiofur against the first three pathogens [11,40], together with the expected antipyretic action of flunixin meglumine. However, because flunixin meglumine was administered only during the initial 1–2 days of treatment and its antipyretic effect is relatively short-lived (approximately ≤ 30 h) [41], the sustained improvement in RT observed through Day 5 is more likely attributable to clinical recovery following antimicrobial therapy, although an early contribution from flunixin meglumine cannot be excluded.

Improvement in RR is consistent with previous reports demonstrating reduced lung consolidation and improved pulmonary function following antimicrobial treatment [42,43]. Recovery of RCR likely reflects restoration of appetite and ruminal motility as systemic illness resolves [44]. Similarly, improvements in CAS corroborate overall clinical recovery and align with earlier BRDC studies using standardized scoring systems [28,45].

From a practical perspective, these findings support the use of tulathromycin for non-lactating cattle due to its long-acting properties, while ceftiofur remains advantageous in lactating cows because of its shorter milk withdrawal period [38,39].

Tulathromycin is a time-dependent bacteriostatic macrolide that inhibits protein synthesis by binding to the 50S ribosomal subunit and shows marked accumulation and prolonged persistence in lung tissue. Peak plasma concentrations occur rapidly after administration, and therapeutic concentrations against BRD-associated pathogens may be maintained for up to 10 days, with an elimination half-life of approximately 2.75 days in plasma and 8.75 days in lung tissue. In contrast, ceftiofur is a time-dependent bactericidal cephalosporin that inhibits bacterial cell wall synthesis. Peak concentrations are reached within a few hours after IM or SC administration, and the elimination half-life ranges from approximately 29 to 33 h. Importantly, ceftiofur residues in milk decline rapidly and may fall below the tolerance level within several hours after the last dose [41]. In the present field study, ceftiofur was administered for 3 consecutive days according to the labeled regimen and routine practice in participating dairy herds, and the dosing duration was not extended to maintain a standardized treatment protocol and to reduce variability under field conditions. Future studies could evaluate whether prolonged ceftiofur regimens provide additional clinical benefits in more severe cases or under different management conditions.

Significant reductions in WBC were observed in both treatment groups from Day 1 to Day 5, indicating resolution of systemic inflammation. Leukocytosis is commonly associated with infection, stress, and dehydration in BRDC-affected cattle [46,47]. Therefore, the observed decline in WBC is consistent with successful antimicrobial intervention.

A significant reduction in Neu was detected only in the tulathromycin-treatment group, although a similar downward trend was observed in the ceftiofur-treatment group. This difference may be attributable to variation in post-treatment median values or to the immunomodulatory effects of macrolides. Tulathromycin has been shown to induce apoptosis of bovine neutrophils and suppress pro-inflammatory cytokine production via inhibition of NF-κB signaling [48], which may explain the more pronounced neutrophil response in this group. Comparable reductions in WBC and Neu following BRDC treatment have been reported previously in cattle recovering from respiratory infection [49].

Significant decreases in RBC, Hb, and HCT were observed in both treatment groups. Elevated RBC indices in BRDC cattle are often associated with hemoconcentration due to dehydration and reduced water intake during acute illness [46]. The observed post-treatment declines likely reflect improved hydration status and normalization of feed and water intake during recovery.

Additionally, chronic respiratory compromise may stimulate compensatory erythropoiesis in response to hypoxia [21]. As pulmonary function improves following effective treatment, this stimulus diminishes, contributing to normalization of RBC-related parameters. Thus, changes in RBC, Hb, and HCT in the present study are consistent with physiological recovery rather than adverse hematological effects of either antibiotic.

Both treatment groups exhibited significant reductions in BUN and Crea from Day 1 to Day 5. Elevated BUN and Crea in BRDC-affected cattle are commonly linked to dehydration, reduced renal perfusion, and catabolic stress [18,21]. The observed declines indicate restoration of hydration and renal function as clinical condition improved. Although previous studies have reported variable effects of BRDC on renal biomarkers [16,17], the consistent reductions observed here further support the conclusion that both antimicrobials facilitated systemic recovery.

Changes in AST, ALT, and ALP varied slightly between treatment groups but generally reflected reduced hepatocellular stress following recovery. Significant reductions in AST and ALT were observed in the ceftiofur-treatment group, whereas ALP declined significantly in the tulathromycin-treatment group. Elevated liver enzymes in BRDC cattle may result from systemic inflammation, muscle exertion due to respiratory distress, and reduced feed intake [18,21]. The post-treatment reductions observed in this study are consistent with improved metabolic and hepatic function following resolution of respiratory disease. Similar enzyme patterns have been reported previously in cattle treated successfully for respiratory infections using macrolide antimicrobials [20,50].

In the metaphylaxis component, no cattle in any of the three groups developed clinical BRDC during the high-PM2.5 period. Therefore, the preventive effect of tulathromycin metaphylaxis on clinical disease could not be determined under the conditions of this study. The GEE results indicated that most hematological and biochemical variables remained broadly stable over the metaphylaxis period, suggesting that pregnant cattle in all regimens maintained overall physiological homeostasis under field conditions. The significant group effect observed for neutrophils, with consistently higher mean values in the no-injection group, may reflect baseline differences in immune tone, gestational stage, or individual variability rather than a clear causal effect of tulathromycin, particularly given the absence of a significant time effect or time × group interaction. Similarly, the divergent patterns of Hb and HCT across groups may be attributable to pregnancy- and hydration-related variation and should be interpreted in the context of signalment and physiological status rather than injection regimen alone. Nevertheless, previous studies have reported associations between particulate matter exposure and changes in Hb and HCT in humans and dairy cattle, potentially linked to systemic inflammation and compensatory erythropoietic responses [22,51]; however, this observation should be interpreted cautiously and cannot be directly inferred from the present study.

The absence of significant changes in key renal markers (BUN and creatinine) across groups and time provides no indication of treatment-associated renal impairment in this cohort. Similarly, ALT remained comparable among regimens and between sampling points, suggesting no evidence of hepatocellular enzyme elevation associated with the tulathromycin injection strategies evaluated. Although statistically significant changes were detected for platelet count over time and for total protein and albumin, these differences were small in magnitude and likely represent expected physiological variation rather than clinically relevant abnormalities. Notably, alterations in total protein and albumin have also been reported under PM2.5 exposure, potentially reflecting inflammatory status, hydration, and hepatic protein synthesis [52]; however, given the multifactorial influences of gestational stage, season, and management, these findings should be interpreted cautiously.

This study was conducted under practical farm conditions during the seasonal period when elevated PM2.5 concentrations are typically observed in northern Thailand. As a result, pathogen identification and antimicrobial susceptibility testing were not routinely feasible prior to treatment. Therefore, the etiological agents responsible for BRDC could not be confirmed, and resistance-related treatment effectiveness could not be evaluated in this cohort. However, the study was not designed to quantify the effect of PM2.5 on BRDC incidence or antimicrobial treatment responses. Importantly, no low-PM2.5 comparison period or contemporaneous control group was included; therefore, no causal inferences can be made regarding the influence of PM2.5 on disease susceptibility, clinical outcomes, or hematological and biochemical changes observed in the present trials. In addition, the relatively small sample size, particularly for between-group comparisons, may have limited sensitivity to detect modest differences across treatment regimens, as enrollment was constrained by feasible case recruitment under routine farm conditions. Furthermore, the absence of clinical BRDC cases in the metaphylaxis component prevented determination of metaphylactic effectiveness under the conditions of this study. Future studies incorporating longitudinal PM2.5 exposure measurements and comparisons across low- and high-PM2.5 periods, together with pathogen, antimicrobial susceptibility testing, and larger sample sizes, are warranted to elucidate the relationship between ambient air pollution and BRDC risk and treatment responses in dairy cattle.

## 4. Materials and Methods

### 4.1. Study Design

A prospective field study was conducted from February to June 2025, corresponding to the annual high-PM2.5 period in northern Thailand. Thirty dairy herds located in Mae Wang, San Pa Tong, and Doi Lo districts of Chiang Mai Province were enrolled. Farm management practices across participating herds were comparable in terms of housing, feed resources, and feeding protocols. All herds were certified under the Good Agricultural Practice (GAP) standards of the Department of Livestock Development. Environmental conditions during the study period included monthly mean PM2.5 concentrations ranging from 10.0 to 81.2 µg/m^3^, ambient temperatures of 25.8–29.8 °C, and relative humidity between 49.0 and 75.0%. Monthly mean values of PM2.5, ambient temperature, and relative humidity during the study period are provided in Supplementary Table S4.

### 4.2. Study Population and Animal Enrollment

Thirty-eight Holstein–Friesian dairy cattle exhibiting clinical signs consistent with BRDC were enrolled to compare the therapeutic efficacy of tulathromycin and ceftiofur. All animals were female and demonstrated respiratory abnormalities on the first day of examination. Cattle that had received any antibiotic or antipyretic treatment prior to evaluation by the research veterinarians were excluded.

For the metaphylaxis trial, eighty-seven pregnant Holstein-Friesian cattle with gestational ages ranging from 2 to 7 months were enrolled and assigned to one of three metaphylactic groups including no injection, a single tulathromycin injection, and two tulathromycin injections administered at a one-month interval. The study was conducted over a three-month period. During the first month, none of the cattle received tulathromycin. In the second month, tulathromycin was administered to 29 cattle in the single-injection group and 29 cattle in the two-injection group. Finally, in the third month, the second dose of tulathromycin was administered to the 29 cattle in the two-injection group. Animals were excluded if they received antibiotic or antipyretic treatment at any point before or during the study, or if any clinical illness was detected during the study period.

### 4.3. Clinical Examination

To compare the therapeutic efficacy of tulathromycin and ceftiofur, signalment data (animal ID, age, sex, breed, and body weight) were recorded for cattle presenting clinical BRDC signs. Physical examinations were performed for five consecutive days, with Day 1 defined as the day of BRDC diagnosis, initiation of treatment, and the first blood sample collection (baseline). Clinical variables assessed included RT, RCR, RR, lung sounds, dyspnea, coughing, appetite, locomotion (normal, slow movement, ataxia, or recumbency), and nasal/ocular discharge (type and amount). CAS were assigned using a five-point scale (0–4), representing normal, mild, moderate, severe, and moribund status, based on established criteria [28,45], The scoring scheme is summarized in Table 5. For the tulathromycin metaphylaxis trial, all animals underwent a baseline physical examination using the same assessment protocol, and their clinical health was monitored throughout the study period.

### 4.4. Treatments

Cattle diagnosed with BRDC were randomly assigned to receive either tulathromycin or ceftiofur treatment. Tulathromycin (Tulissin^®^, Virbac, Carros, France) was administered subcutaneously at 2.5 mg/kg body weight as a single dose, whereas ceftiofur (Citius^®^, Virbac, Lyon, France) was administered intramuscularly at 2.2 mg/kg body weight once daily for three consecutive days. In both treatment groups, all cattle additionally received flunixin meglumine (Meifluxin^®^, Thai Meiji Pharmaceutical, Bangkok, Thailand) at 2.2 mg/kg IV on Days 1 and 2 as supportive therapy for antipyretic and anti-inflammatory management, according to the manufacturer’s recommendations.

For the metaphylaxis study, cattle were allocated into three groups based on injection regimen including a no-injection group (*n* = 29), a one-time injection group (*n* = 29), and a two-times injection group (*n* = 29). In the injection groups, tulathromycin (Tulissin^®^, Virbac, France) was administered subcutaneously at 2.5 mg/kg body weight as a single-dose injection per administration, with the second injection in the two-times group given 1 month after the first. Body weight was measured using a weight tape prior to each injection to ensure accurate dose calculation.

### 4.5. Blood Collection

Blood samples were intended to be collected from all cattle on Days 1 and 5. However, due to an early sampling/logistical error at study initiation, paired Day 1 and Day 5 blood samples were available for 16 tulathromycin-treated and 15 ceftiofur-treated cattle for hematological and biochemical analyses. Samples were obtained from the coccygeal vein into serum and EDTA tubes and submitted to the laboratory for complete blood count and serum biochemistry analyses, including WBC, Neu, Lymp, RBC, Hb, HCT, BUN, creatinine, AST, ALT, and ALP.

Blood samples were collected from all enrolled cattle at two time points for the tulathromycin metaphylaxis study, including before the injection of tulathromycin in all cattle (first blood collection) and one month interval after the tulathromycin injection in the two-injection group (second blood collection). Blood samples were collected from the coccygeal vein and placed into serum and EDTA tubes. Blood parameters measured included WBC, Neu, Lymp, RBC, Hb, HCT, BUN, Crea, total protein, albumin, and ALT.

### 4.6. Statistical Analysis

For the BRDC treatment comparison, continuous variables (RR, RCR, and RT) were analyzed using the Mann–Whitney U test or two-way repeated measures ANOVA, depending on distribution and model assumptions. Categorical variables (mental status, coughing, nasal discharge, appetite, locomotion, and CAS were analyzed using the Chi-square test or Fisher’s exact test when applicable. Survival analysis was performed using Kaplan–Meier curves to compare mortality over the 5-day follow-up period between treatment groups. Time-to-event was defined as the last day of observation, and animals that survived to Day 5 were treated as censored observations. On the other hand, within-group changes from day 1 to day 5 were assessed using the Wilcoxon signed-rank test for continuous variables and the McNemar test for categorical outcomes.

For the metaphylaxis study, generalized estimating equations (GEE) were used to evaluate differences between the first and second blood collections across the three injection groups. Factors included sampling time, injection type, and their interaction. Significance for all analyses was defined at *p* < 0.05.

## 5. Conclusions

This field study demonstrates that tulathromycin and ceftiofur provide comparable therapeutic efficacy for the treatment of undifferentiated BRDC in dairy cattle under practical farm conditions. Both antimicrobials resulted in significant clinical and hematological improvement within 5 days, with similar cure and case fatality rates, supporting their use when etiological confirmation is not immediately feasible. From a clinical management perspective, tulathromycin may be advantageous in non-lactating cattle due to its long-acting properties, whereas ceftiofur remains suitable for lactating cows due to its zero milk withdrawal time according to label recommendations, minimizing milk discard. In the metaphylaxis component conducted during the high-PM2.5 season, no clinical BRDC cases were observed across treatment groups, precluding assessment of preventive efficacy. Although selected hematological and biochemical differences were observed among injection groups during this period, these findings are descriptive and should be interpreted cautiously, as they may reflect physiological variation and potential confounding effects of gestational stage, seasonal conditions, hydration status, and herd management rather than a direct effect of PM2.5 exposure or tulathromycin administration. Future studies incorporating larger populations, pathogen detection, antimicrobial susceptibility testing, and quantitative PM2.5 exposure assessment are necessary to refine evidence-based BRDC prevention and treatment strategies in dairy herds.

## Figures and Tables

**Table 1:** Baseline demographic, clinical, and physiological characteristics of dairy cattle with undifferentiated bovine respiratory disease complex (BRDC) treated with tulathromycin or ceftiofur.

Parameter	Tulathromycin	Ceftiofur	*p*-Value
Number of animals (head)	20	18	
Age (months)	11.2 ± 8.1	21.7 ± 16.6	*p* > 0.05
Body weight (kg)	203.1 ± 109.8	300.2 ± 150.3	*p* > 0.05
Rectal temperature (°F)	104.2 ± 1.6	104.0 ± 1.6	*p* > 0.05
Respiratory rate (time/min)	82.5 ± 22.5	72.5 ± 17.1	*p* > 0.05
Ruminal contraction (time/2 min)	0.3 ± 0.8	0.5 ± 1.0	*p* > 0.05
Clinical attitude score	2.9 ± 0.7	2.9 ± 0.2	*p* > 0.05
Fever (%)	80.0	72.2	*p* > 0.05
Nasal discharge (%)	95.0	100.0	*p* > 0.05
Coughing (%)	30.0	22.2	*p* > 0.05
Anorexia (%)	100.0	94.4	*p* > 0.05
Abnormal locomotion (%)	90.0	100.0	*p* > 0.05

**Table 2:** Within-group changes in clinical parameters between Day 1 and Day 5 in dairy cattle with undifferentiated bovine respiratory disease complex (BRDC) treated with tulathromycin or ceftiofur.

Group	Parameter	Day 1	Day 5	*n*	*p*-Value
Tulathromycin	Rectal temperature (°F)	104.4 ± 1.5	102.1 ± 1.2	18	0.002
Tulathromycin	Respiratory rate (times/min)	82.8 ± 23.8	54.4 ± 26.8	18	0.001
Tulathromycin	Ruminal contraction (times/2 min)	0.4 ± 0.9	3.0 ± 0.0	18	<0.001
Tulathromycin	Clinical attitude score	2.8 ± 0.7	0.2 ± 0.4	18	<0.001
Ceftiofur	Rectal temperature (°F)	104.2 ± 1.5	102.0 ± 1.4	16	0.001
Ceftiofur	Respiratory rate (times/min)	70.4 ± 17.1	47.2 ± 16.4	16	0.001
Ceftiofur	Ruminal contraction (times/2 min)	0.6 ± 1.0	2.9 ± 0.2	16	<0.001
Ceftiofur	Clinical attitude score	2.9 ± 0.2	0.4 ± 0.7	16	<0.001

**Table 3:** Within-group changes in hematological and biochemical parameters between Day 1 and Day 5 in dairy cattle with undifferentiated bovine respiratory disease complex (BRDC) treated with tulathromycin or ceftiofur.

Group	Parameter	Day 1	Day 5	*n*	*p*-Value
Tulathromycin	WBC (×10^9^/L) ^1^	14.1 ± 6.0	11.3 ± 2.3	16	0.036
Tulathromycin	Neutrophils	8.7 ± 4.3	5.7 ± 2.2	16	0.029
Tulathromycin	Lymphocytes	5.1 ± 1.8	5.2 ± 1.5	16	0.698
Tulathromycin	RBC (×10^12^/L) ^2^	8.1 ± 1.3	7.3 ± 1.2	16	<0.001
Tulathromycin	Hemoglobin (g/dL)	10.0 ± 1.5	8.8 ± 1.1	16	0.005
Tulathromycin	Hematocrit (%)	29.4 ± 3.9	26.6 ± 3.1	16	0.001
Tulathromycin	BUN (mg/dL) ^3^	6.9 ± 3.8	4.4 ± 3.0	16	0.008
Tulathromycin	Creatinine (mg/dL)	1.1 ± 0.2	1.0 ± 0.1	16	0.038
Tulathromycin	AST (U/L) ^4^	88.1 ± 27.7	82.1 ± 30.9	16	0.394
Tulathromycin	ALP (U/L) ^5^	130.1 ± 72.4	96.4 ± 26.1	16	0.029
Tulathromycin	ALT (U/L) ^6^	20.0 ± 8.2	17.8 ± 5.6	16	0.262
Ceftiofur	WBC (×10^9^/L)	14.5 ± 5.1	11.8 ± 3.5	15	0.041
Ceftiofur	Neutrophils	8.5 ± 3.6	6.1 ± 2.5	15	0.083
Ceftiofur	Lymphocytes	5.3 ± 2.8	5.2 ± 2.9	15	0.513
Ceftiofur	RBC (×10^12^/L)	7.4 ± 1.1	6.9 ± 1.2	15	0.012
Ceftiofur	Hemoglobin (g/dL)	9.7 ± 1.0	8.8 ± 1.3	15	0.004
Ceftiofur	Hematocrit (%)	28.3 ± 2.8	26.5 ± 3.2	15	0.012
Ceftiofur	BUN (mg/dL)	9.3 ± 5.7	6.2 ± 4.3	15	0.030
Ceftiofur	Creatinine (mg/dL)	1.2 ± 0.1	1.1 ± 0.1	15	0.005
Ceftiofur	AST (U/L)	81.2 ± 21.7	68.6 ± 16.3	15	0.033
Ceftiofur	ALP (U/L)	92.4 ± 50.1	82.9 ± 35.0	15	0.243
Ceftiofur	ALT (U/L)	22.3 ± 12.4	14.7 ± 5.0	15	0.003

^1^ WBC, white blood cell count; ^2^ RBC, red blood cell count; ^3^ BUN, blood urea nitrogen; ^4^ AST, aspartate aminotransferase; ^5^ ALP, alkaline phosphatase; ^6^ ALT, alanine aminotransferase. Note: Hematological and biochemical analyses were performed on cattle with paired Day 1 and Day 5 blood samples available (tulathromycin, *n* = 16; ceftiofur, *n* = 15).

**Table 4:** Hematological and serum biochemical responses before and after tulathromycin metaphylaxis under field conditions in dairy cattle, analyzed by generalized estimating equation (GEE).

Parameter	Time ^1^	No Injection ^2^	One Injection ^3^	Two Injections ^4^	*p*-Value (Group) ^5^	*p*-Value (Time) ^6^	*p*-Value (Time × Group) ^7^
Neutrophils	before ^8^	4.5 ± 1.4	4.2 ± 1.4	3.6 ± 1.2	0.01	0.96	0.61
Neutrophils	after ^9^	4.5 ± 1.8	3.8 ± 1.3	3.6 ± 1.2			
Hemoglobin (g/dL)	before	10.6 ± 0.8	10.6 ± 1.1	10.9 ± 1.7	<0.001	0.55	0.001
Hemoglobin (g/dL)	after	10.0 ± 0.8	10.7 ± 1.0	11.4 ± 0.9			
Hematocrit (%)	before	35.1 ± 2.2	34.6 ± 3.3	35.9 ± 5.1	<0.001	0.50	<0.001
Hematocrit (%)	after	33.3 ± 2.3	35.5 ± 3.0	37.5 ± 2.6			
Platelet (×10^3^/µL) ^2^	before	215.9 ± 113.5	274.9 ± 86.5	238.7 ± 94.1	0.07	0.002	0.12
Platelet (×10^3^/µL) ^2^	after	283.0 ± 122.1	287.2 ± 106.2	257.1 ± 96.0			
Albumin (g/dL)	before	3.5 ± 0.3	3.4 ± 0.2	3.5 ± 0.2	0.06	0.04	0.83
Albumin (g/dL)	after	3.4 ± 0.2	3.4 ± 0.2	3.5 ± 0.2			

^1^ Time—time for blood collection; ^2^ No injection—no tulathromycin metaphylaxis administered; ^3^ One injection—a single dose administered on Day 0; ^4^ Two injections—doses administered on Day 0 and Day 30.; ^5^ *p*-value (Group)—the main effect of injection regimen; ^6^ *p*-value (Time)—the main effect of sampling time (before vs. after injections); ^7^ *p*-value (Time × Group)—the interaction effect between injection regimen and time; ^8^ before—blood sampling prior to tulathromycin metaphylaxis; ^9^ after—blood sampling 30 days after the final injection.

**Table 5:** Clinical attitude score (CAS) system used to evaluate the severity of clinical signs in dairy cattle affected by bovine respiratory disease complex (BRDC).

CAS	Diagnosis	Clinical Signs
0	Normal	Bright, alert, and responsive
1	Mild	Brightens, moves readily, and appears normal May be mildly depressed and a small amount of nasal or ocular discharge may be present
2	Moderate	Does not brighten up and move slowly Moderate depressed and may exhibit dyspnea, a considerable nasal or ocular discharge, and coughing
3	Severe	Stumbles or moves only with extreme coercion Severely depressed and may be anorexic and exhibit coughing with copious nasal discharge
4	Moribund	Recumbent and not able or willing to rise or take food and/or water

## Data Availability

The data supporting the findings of this study are available from the corresponding author upon reasonable request.

## Supplementary Materials

The following supporting information can be downloaded at: https://www.mdpi.com/article/10.3390/antibiotics15020154/s1, Tables S1–S4: Clinical characteristics, hematological and biochemical parameters, metaphylaxis responses, and environmental data.

## Abbreviations

ALP	Alkaline phosphatase
ALT	Alanine aminotransferase
AST	Aspartate aminotransferase
BRDC	Bovine respiratory disease complex
BUN	Blood urea nitrogen
CAS	Clinical attitude score
GEE	Generalized estimating equations
GAP	Good Agricultural Practice
Hb	Hemoglobin
HCT	Hematocrit
*H. somni*	*Histophilus somni*
Lymp	Lymphocytes
*M. bovis*	*Mycoplasma bovis*
*M. haemolytica*	*Mannheimia haemolytica*
Neu	Neutrophils
*P. multocida*	*Pasteurella multocida*
PM2.5	Particulate matter with aerodynamic diameter ≤ 2.5 µm
RCR	Ruminal contraction rate
RR	Respiratory rate
RT	Rectal temperature
RBC	Red blood cell count
WBC	White blood cell count
