# Supplementary material for: Comparative Efficacy of Tulathromycin and Ceftiofur for Treating Undifferentiated BRDC and Tulathromycin Metaphylaxis in Dairy Cattle
Source: Antibiotics (Basel). 2026 Feb 2;15(2):154. doi: 10.3390/antibiotics15020154 (PMC12937335; doi:10.3390/antibiotics15020154)
Supplement: Supplementary file 1 [file antibiotics-15-00154-s001.zip › antibiotics-4099717-supplementary.pdf]

Supplementary Table S1.1 Comparison of baseline physical examination findings of dairy cattle with undifferentiated bovine respiratory disease complex (BRDC) between tulathromycin and ceftiofur treatment groups on Day 1.

Parameter	Tulathromycin	Ceftiofur	p-value
Number of animals (head)	20	18	
Age (months)	11.2 ± 8.1	21.7 ± 16.6	$p > 0.05$
Body weight (kg)	203.1 ± 109.8	300.2 ± 150.3	$p > 0.05$
Rectal temperature (°F)	104.2 ± 1.6	104.0 ± 1.6	$p > 0.05$
Respiratory rate (time/min)	82.5 ± 22.5	72.5 ± 17.1	$p > 0.05$
Ruminal contraction (time/ 2 min)	0.3 ± 0.8	0.5 ± 1.0	$p > 0.05$
Clinical attitude score	2.9 ± 0.7	2.9 ± 0.2	$p > 0.05$
Fever (%)	80.0	72.2	$p > 0.05$
Nasal discharge (%)	95.0	100.0	$p > 0.05$
Coughing (%)	30.0	22.2	$p > 0.05$
Anorexia (%)	100.0	94.4	$p > 0.05$
Abnormal locomotion (%)	90.0	100.0	$p > 0.05$

Supplementary Table S1.2 Comparison of clinical and physiological characteristics of dairy cattle with undifferentiated bovine respiratory disease complex (BRDC) treated with tulathromycin or ceftiofur on Day 2.

Parameter	Tulathromycin	Ceftiofur	p-value
Number of animals (head)	19	17	
Rectal temperature (°F)	103.4 ± 2.5	102.8 ± 1.4	$p > 0.05$
Respiratory rate (time/min)	71.3 ± 24.8	60.4 ± 22.9	$p > 0.05$
Ruminal contraction (time/ 2 min)	2.0 ± 1.2	2.2 ± 1.1	$p > 0.05$
Clinical attitude score	1.7 ± 0.9	1.3 ± 0.6	$p > 0.05$
Fever (%)	68.4	47.1	$p > 0.05$
Nasal discharge (%)	15.7	17.6	$p > 0.05$
Coughing (%)	21.1	11.8	$p > 0.05$
Anorexia (%)	26.3	17.6	$p > 0.05$
Abnormal locomotion (%)	57.9	41.2	$p > 0.05$

Supplementary Table S1.3 Comparison of clinical and physiological characteristics of dairy cattle with undifferentiated bovine respiratory disease complex (BRDC) treated with tulathromycin or ceftiofur on Day 3.

Parameter	Tulathromycin	Ceftiofur	p-value
Number of animals (head)	18	16	
Rectal temperature (°F)	101.9 ± 1.0	102.2 ± 1.3	p > 0.05
Respiratory rate (time/min)	55.5 ± 21.9	58.2 ± 19.9	p > 0.05
Ruminal contraction (time/ 2 min)	2.9 ± 0.4	2.7 ± 0.6	p > 0.05
Clinical attitude score	1.1 ± 0.7	1.0 ± 0.5	p > 0.05
Fever (%)	11.1	22.2	p > 0.05
Nasal discharge (%)	47.1	62.5	p > 0.05
Coughing (%)	16.7	12.5	p > 0.05
Anorexia (%)	5.6	0	p > 0.05
Abnormal locomotion (%)	11.1	6.3	p > 0.05

Supplementary Table S1.4 Comparison of clinical and physiological characteristics of dairy cattle with undifferentiated bovine respiratory disease complex (BRDC) treated with tulathromycin or ceftiofur on Day 4.

Parameter	Tulathromycin	Ceftiofur	p-value
Number of animals (head)	18	16	
Rectal temperature (°F)	102.0 ± 1.2	102.2 ± 1.2	p > 0.05
Respiratory rate (time/min)	53.4 ± 18.74	51.8 ± 22.5	p > 0.05
Ruminal contraction (time/ 2 min)	3.0 ± 0.4	2.75 ± 0.6	p > 0.05
Clinical attitude score	0.3 ± 0.6	0.4 ± 0.6	p > 0.05
Fever (%)	16.7	22.2	p > 0.05
Nasal discharge (%)	11.1	6.3	p > 0.05
Coughing (%)	11.1	6.3	p > 0.05
Anorexia (%)	0	0	p > 0.05
Abnormal locomotion (%)	0	12.5	p > 0.05

Supplementary Table S1.5 Comparison of clinical and physiological characteristics of dairy cattle with undifferentiated bovine respiratory disease complex (BRDC) treated with tulathromycin or ceftiofur on Day 5.

Parameter	Tulathromycin	Ceftiofur	p-value
Number of animals (head)	18	16	
Rectal temperature (°F)	102.1 ± 1.2	101.9 ± 1.4	$p > 0.05$
Respiratory rate (time/min)	54.4 ± 26.8	47.25 ± 16.4	$p > 0.05$
Ruminal contraction (time/ 2 min)	3.0 ± 0.0	2.9 ± 0.2	$p > 0.05$
Clinical attitude score	0.2 ± 0.4	0.4 ± 0.7	$p > 0.05$
Fever (%)	22.2	31.3	$p > 0.05$
Nasal discharge (%)	0	6.3	$p > 0.05$
Coughing (%)	11.1	12.5	$p > 0.05$
Anorexia (%)	0	0	$p > 0.05$
Abnormal locomotion (%)	0	12.5	$p > 0.05$

Supplementary Table S2.1 Between-group comparisons of hematological and biochemical parameters at Day 1 in dairy cattle with undifferentiated bovine respiratory disease complex (BRDC) treated with tulathromycin or ceftiofur.

Parameter	Tulathromycin	Ceftiofur	p-value
Number of animals (head)	16	15	
WBC ($\times 10^9$/L)[1]	14.1 ± 6.0	14.5 ± 5.1	$p > 0.05$
Neutrophils	8.7 ± 4.3	8.5 ± 3.6	$p > 0.05$
Lymphocytes	5.1 ± 1.8	5.3 ± 2.8	$p > 0.05$
RBC ($\times 10^{12}$/L)[2]	8.1 ± 1.3	7.4 ± 1.1	$p > 0.05$
Hemoglobin (g/dL)	10.0 ± 1.5	9.7 ± 1.0	$p > 0.05$
Hematocrit (%)	29.4 ± 3.9	28.3 ± 2.8	$p > 0.05$
BUN (mg/dL)[3]	6.9 ± 3.8	9.3 ± 5.7	$p > 0.05$
Creatinine (mg/dL)	1.1 ± 0.2	1.2 ± 0.1	$p > 0.05$
AST (U/L)[4]	88.1 ± 27.7	81.2 ± 21.7	$p > 0.05$
ALP (U/L)[5]	130.1 ± 72.4	92.4 ± 50.1	$p > 0.05$
ALT (U/L)[6]	20.0 ± 8.2	22.3 ± 12.4	$p > 0.05$

[1]WBC, white blood cell count; [2]RBC, red blood cell count; [3]BUN, blood urea nitrogen; [4]AST, aspartate aminotransferase; [5]ALP, alkaline phosphatase; [6]ALT, alanine aminotransferase.

Supplementary Table S1.2 Between-group comparisons of hematological and biochemical parameters at Day 5 in dairy cattle with undifferentiated bovine respiratory disease complex (BRDC) treated with tulathromycin or ceftiofur.

Parameter	Tulathromycin	Ceftiofur	p-value
Number of animals (head)	16	15	
WBC ($\times 10^9$/L)[1]	11.3 ± 2.3	11.8 ± 3.5	$p > 0.05$
Neutrophils	5.7 ± 2.2	6.1 ± 2.5	$p > 0.05$
Lymphocytes	5.2 ± 1.5	5.2 ± 2.9	$p > 0.05$
RBC ($\times 10^{12}$/L)[2]	7.3 ± 1.2	6.9 ± 1.2	$p > 0.05$
Hemoglobin (g/dL)	8.8 ± 1.1	8.8 ± 1.3	$p > 0.05$
Hematocrit (%)	26.6 ± 3.1	26.5 ± 3.2	$p > 0.05$
BUN (mg/dL)[3]	4.4 ± 3.0	6.2 ± 4.3	$p > 0.05$
Creatinine (mg/dL)	1.0 ± 0.1	1.1 ± 0.1	$p > 0.05$
AST (U/L)[4]	82.1 ± 30.9	68.6 ± 16.3	$p > 0.05$
ALP (U/L)[5]	96.4 ± 26.1	82.9 ± 35.0	$p > 0.05$
ALT (U/L)[6]	17.8 ± 5.6	14.7 ± 5.0	$p > 0.05$

[1]WBC, white blood cell count; [2]RBC, red blood cell count; [3]BUN, blood urea nitrogen; [4]AST, aspartate aminotransferase; [5]ALP, alkaline phosphatase; [6]ALT, alanine aminotransferase.

Supplementary Table S3. Hematological and serum biochemical responses before and after tulathromycin metaphylaxis under field conditions in dairy cattle, analyzed by generalized estimating equation (GEE).

Parameter	Time[1]	no-injection[2]	one-injection[3]	two-injection[4]	p-value (Group)[5]	p-value (Time)[6]	p-value (Time×Group)[7]
WBC ($\times 10^9$/L)[1]	before[8]	14.7 ± 5.7	13.1 ± 4.0	13.5 ± 7.6	0.49	0.11	0.60
WBC ($\times 10^9$/L)[1]	after[9]	13.6 ± 7.7	11.9 ± 3.8	13.4 ± 10.4			
Neutrophils	before	4.5 ± 1.4	4.2 ± 1.4	3.6 ± 1.2	0.01	0.96	0.61
Neutrophils	after	4.5 ± 1.8	3.8 ± 1.3	3.6 ± 1.2			
Lymphocytes	before	8.5 ± 4.2	7.5 ± 3.5	8.6 ± 6.4	0.57	0.29	0.51
Lymphocytes	after	7.7 ± 6.7	6.7 ± 2.6	8.7 ± 9.1			
Monocytes	before	1.4 ± 1.1	1.1 ± 0.5	1.0 ± 0.5	0.14	0.07	0.51
Monocytes	after	1.2 ± 0.8	1.0 ± 0.6	0.7 ± 0.4			
Eosinophils	before	0.3 ± 0.4	0.4 ± 0.2	0.3 ± 0.3	0.97	0.22	0.26
Eosinophils	after	0.2 ± 0.2	0.4 ± 0.3	0.4 ± 0.2			
RBC ($\times 10^{12}$/L)[2]	before	7.2 ± 0.7	7.2 ± 0.8	7.2 ± 1.1	0.17	0.42	0.07
RBC ($\times 10^{12}$/L)[2]	after	6.7 ± 0.6	7.1 ± 0.6	7.2 ± 0.6			
Hemoglobin (g/dL)	before	10.6 ± 0.8	10.6 ± 1.1	10.9 ± 1.7	<0.001	0.55	0.001
Hemoglobin (g/dL)	after	10.0 ± 0.8	10.7 ± 1.0	11.4 ± 0.9			
Hematocrit (%)	before	35.1 ± 2.2	34.6 ± 3.3	35.9 ± 5.1	<0.001	0.50	<0.001
Hematocrit (%)	after	33.3 ± 2.3	35.5 ± 3.0	37.5 ± 2.6			

[1]Time, time for blood collection; [2]No-injection, no tulathromycin metaphylaxis administered; [3]One-injection, a single dose administered on Day 0; [4]Two-injection, doses administered on Day 0 and Day 30.; [5]p-value (Group), the main effect of injection regimen; [6]p-value (Time), the main effect of sampling time (before vs after injections); [7]p-value (Time×Group), the interaction effect between injection regimen and time; [8]before, blood sampling prior to tulathromycin metaphylaxis; [9]after, blood sampling 30 days after the final injection.

Supplementary Table S3. Hematological and serum biochemical responses before and after tulathromycin metaphylaxis under field conditions in dairy cattle, analyzed by generalized estimating equation (GEE) (cont.).

Parameter	Time[1]	no-injection[2]	one-injection[3]	two-injection[4]	p-value (Group)[5]	p-value (Time)[6]	p-value (Time×Group)[7]
Platelet (×10³/μL)[2]	before[8]	215.9 ± 113.5	274.9 ± 86.5	238.7 ± 94.1	0.07	0.002	0.12
Platelet (×10³/μL)[2]	after[9]	283.0 ± 122.1	287.2 ± 106.2	257.1 ± 96.0			
BUN (mg/dL)[3]	before	5.6 ± 2.9	5.0 ± 2.2	6.2 ± 3.3	0.22	0.22	0.63
BUN (mg/dL)[3]	after	6.3 ± 3.8	5.3 ± 2.6	6.0 ± 3.0			
Creatinine (mg/dL)	before	1.5 ± 0.3	1.4 ± 0.2	1.4 ± 0.2	0.11	0.08	0.14
Creatinine (mg/dL)	after	1.4 ± 0.2	1.4 ± 0.2	1.4 ± 0.1			
Total Protein (g/dL)	before	7.8 ± 0.7	7.6 ± 0.5	7.8 ± 0.5	0.11	0.35	0.006
Total Protein (g/dL)	after	8.0 ± 0.8	7.4 ± 0.7	7.5 ± 0.7			
Albumin (g/dL)	before	3.5 ± 0.3	3.4 ± 0.2	3.5 ± 0.2	0.06	0.04	0.83
Albumin (g/dL)	after	3.4 ± 0.2	3.4 ± 0.2	3.5 ± 0.2			
ALT (U/L)[6]	before	21.2 ± 4.9	21.7 ± 4.5	23.4 ± 3.4	0.08	0.07	0.57
ALT (U/L)[6]	after	19.6 ± 4.0	21.7 ± 6.1	22.7 ± 4.7			

[1]Time, time for blood collection; [2]No-injection, no tulathromycin metaphylaxis administered; [3]One-injection, a single dose administered on Day 0; [4]Two-injection, doses administered on Day 0 and Day 30.; [5]p-value (Group), the main effect of injection regimen; [6]p-value (Time), the main effect of sampling time (before vs after injections); [7]p-value (Time×Group), the interaction effect between injection regimen and time; [8]before, blood sampling prior to tulathromycin metaphylaxis; [9]after, blood sampling 30 days after the final injection.

Supplementary Table S4. Monthly mean environmental parameters during the study period (Feb–Jun 2025) in Chiang Mai, Thailand.

Month (2025)	PM2.5 (μg/m³)	Ambient temperature (°C)	Relative humidity (%)
February	45.0	25.8	55.0
March	81.2	27.5	49.0
April	68.3	29.8	54.0
May	26.5	28.5	69.0
June	10.0	28.8	75.0

Note: Monthly mean PM2.5 ($\mu g/m^3$), ambient temperature (°C), and relative humidity (%) were obtained from the Climate Change Data Center, Chiang Mai University (CMUCCDC) Air Quality Information portal (https://www.cmuccdc.org/air-quality-information; accessed 23 October 2025).
